# Supplementary material for: An improved animal model for herpesvirus encephalitis in humans
Source: PLoS Pathog. 2020 Mar 30;16(3):e1008445. doi: 10.1371/journal.ppat.1008445 (PMC7145201; doi:10.1371/journal.ppat.1008445)
Supplement: S1 Table — Three categories including (I) external appearance, (II) behavior and activity and (III) body weight were assessed daily and utilized to group mice into either mildly (max. score 1 in three out of three categories), moderate (max. score 2 in two out of three categories) and severely affected (max. score 2 in all categories or max. score 3 in one out of three categories). (DOCX) [file ppat.1008445.s001.docx]

| **Category** | **Alteration** | **Score** |
| --- | --- | --- |
| I: External appearance | Normal: smooth and glossy fur, no pruritus | 0 |
|  | Normal posture; ruffled and dull fur; mild pruritus; nasal edema | 1 |
|  | Slightly curved back; ruffled and dull fur; moderate pruritus; dermal erosions | 2 |
|  | Severely curved back; ruffled, dull fur; severe pruritus; automutilation; dyspnea; seizures | 3 |
| II: Behavior and activity | Intently and curious | 0 |
|  | Very calm; mild reduced spontaneous activity; no reduced induced activity, photophobia, stargazing | 1 |
|  | Paroxysmal hyperactivity; apathy; moderate reduced spontaneous activity; mild reduced induced activity, fasciculations | 2 |
|  | Stupor; no spontaneous activity; lateral position | 3 |
| III: Body weight | Reduction > 5% | 0 |
|  | Reduction 5-10% | 1 |
|  | Reduction 10-15% | 2 |
|  | Reduction 20% | 3 |
