# Supplementary material for: An improved animal model for herpesvirus encephalitis in humans
Source: PLoS Pathog. 2020 Mar 30;16(3):e1008445. doi: 10.1371/journal.ppat.1008445 (PMC7145201; doi:10.1371/journal.ppat.1008445)
Supplement: S2 Table — The onset of disease, MTD as well as typical clinical signs, which have been observed in mice infected with different virus mutants are listed.— = not present, ✓ = present, (✓) = occasionally observed. (DOCX) [file ppat.1008445.s002.docx]

|  |  | **PrV-Ka** | **PrV-US3∆kin** | **PrV-∆UL21** | **PrV-∆UL21/US3∆kin** |
| --- | --- | --- | --- | --- | --- |
|  | Onset of clinical signs (h p.i.) | 47 ± 7 | 51 ± 12 | 96 ± 0 | 152 ± 27 |
|  | MTD (h p.i.) | 62 ± 5 | 63 ± 5 | 110 ± 8.5 | 1/6 ꝉ |
| Clinical signs | Conjunctivitis | ✓ | ✓ | ✓ | (✓) |
|  | Nasal bridge edema | ✓ | ✓ | ✓ | ✓ |
|  | Pruritus | ✓ | ✓ | ✓ | ✓ |
|  | Hunching | ✓ | ✓ | ✓ | ✓ |
|  | Ruffled fur | ✓ | ✓ | ✓ | ✓ |
|  | Hemorrhagic skin erosions | ✓ | ✓ | ✓ | - |
|  | Automutilation | ✓ | ✓ | ✓ | - |
|  | Dyspnea | ✓ | ✓ | ✓ | - |
|  | Apathy | ✓ | ✓ | ✓ | (✓) |
|  | Hairless skin lesions | - | - | - | ✓ |
|  | Behavioral impairment (e.g. stargazing, slow movements) | - | - | - | ✓ |
|  | Seizures | - | - | - | (✓) |
