# Supplementary figures and images for: An improved animal model for herpesvirus encephalitis in humans
Source: PLoS Pathog. 2020 Mar 30;16(3):e1008445. doi: 10.1371/journal.ppat.1008445 (PMC7145201; doi:10.1371/journal.ppat.1008445)

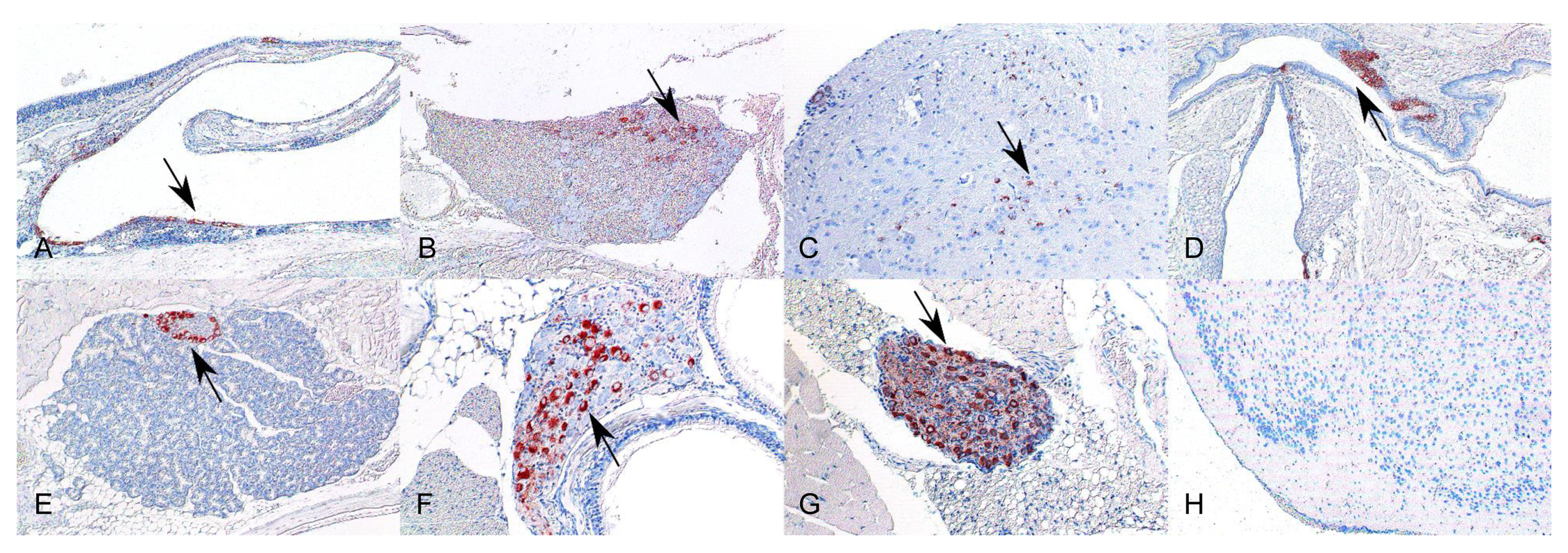

Supplement: S1 Fig — Immunohistochemistry using an anti-glycoprotein-B-antibody was performed to detect PrV in the respective tissues. A) nasal respiratory epithelium (RE), B) trigeminal ganglion (TG), C) spinal trigeminal nucleus (Sp5), D) nasopharynx (NP), E) salivary glands (SG), F) pterygopalatine ganglion (PtG), G) superior cervical ganglion (SCG) and H) cerebral cortex. Viral antigen has not been detected in the cerebral cortex. (TIF) [file ppat.1008445.s003.tif]

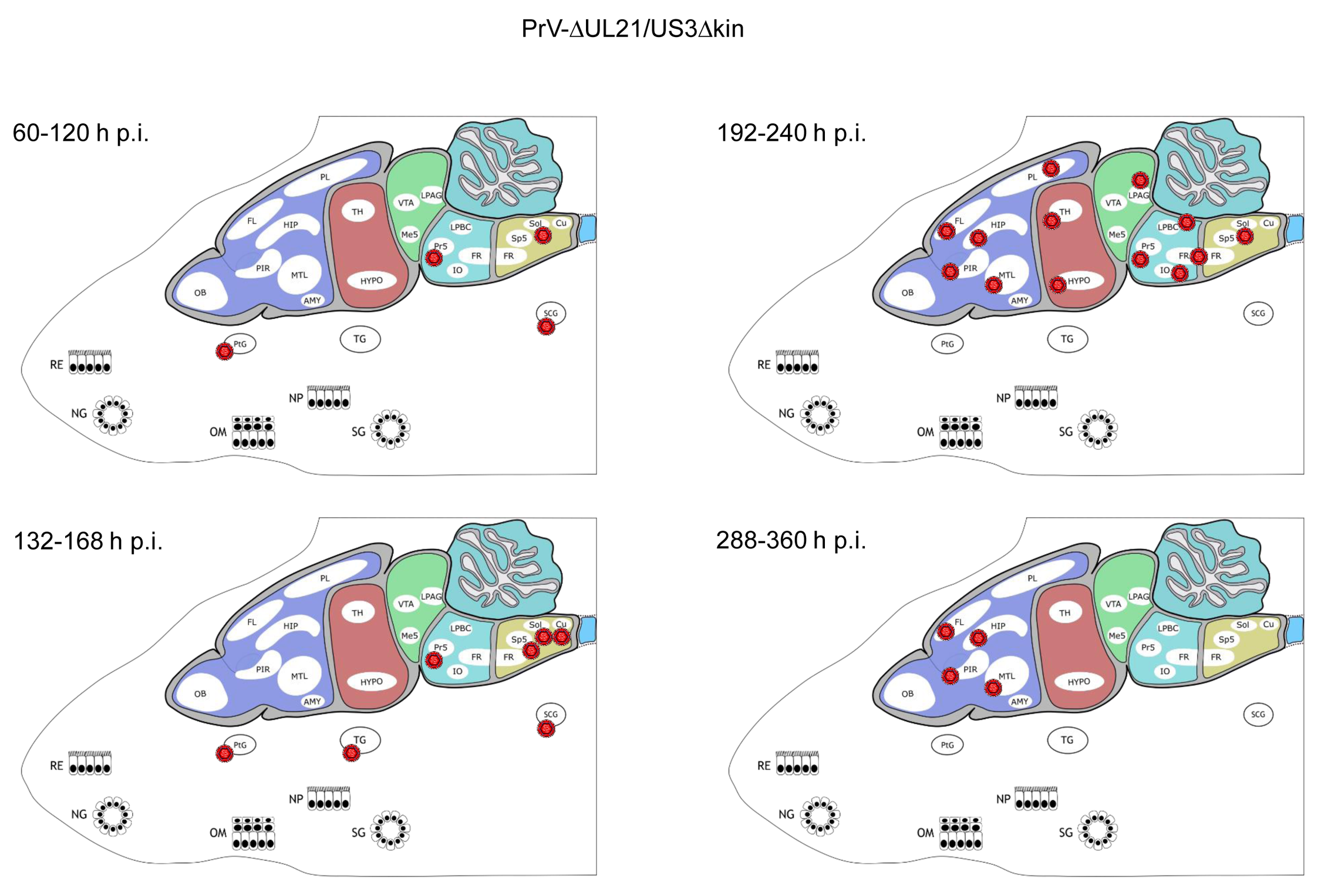

Supplement: S2 Fig — Localization of viral antigen detection is shown for different times p.i. No viral antigen was detectable later than 360 h p.i. RE = respiratory epithelium, NG = nasal gland, OM = oral mucosa, NP = nasopharynx, SG = salivary gland, TG = trigeminal ganglion, PtG = pterygopalatine ganglion, SCG = superior cervical ganglion, Cu = cuneate nucleus, Sol = solitary tract, Sp5 = spinal trigeminal nucleus, FR = reticular formation, IO = inferior olive, LPBC = lateral parabrachial nucleus, Pr5 = principal sensory trigeminal nucleus, Me5 = mesencephalic trigeminal nucleus, PAG = periaqueductal grey, VTA = ventral tegmental area, TH = thalamus, HYPO = hypothalamus, OB = olfactory bulb, FL = frontal lobe, PL = parietal lobe, PIR = piriform lobe, MTL = mesiotemporal lobe, HIP = hippocampus, AMY = amygdala. (TIF) [file ppat.1008445.s004.tif]

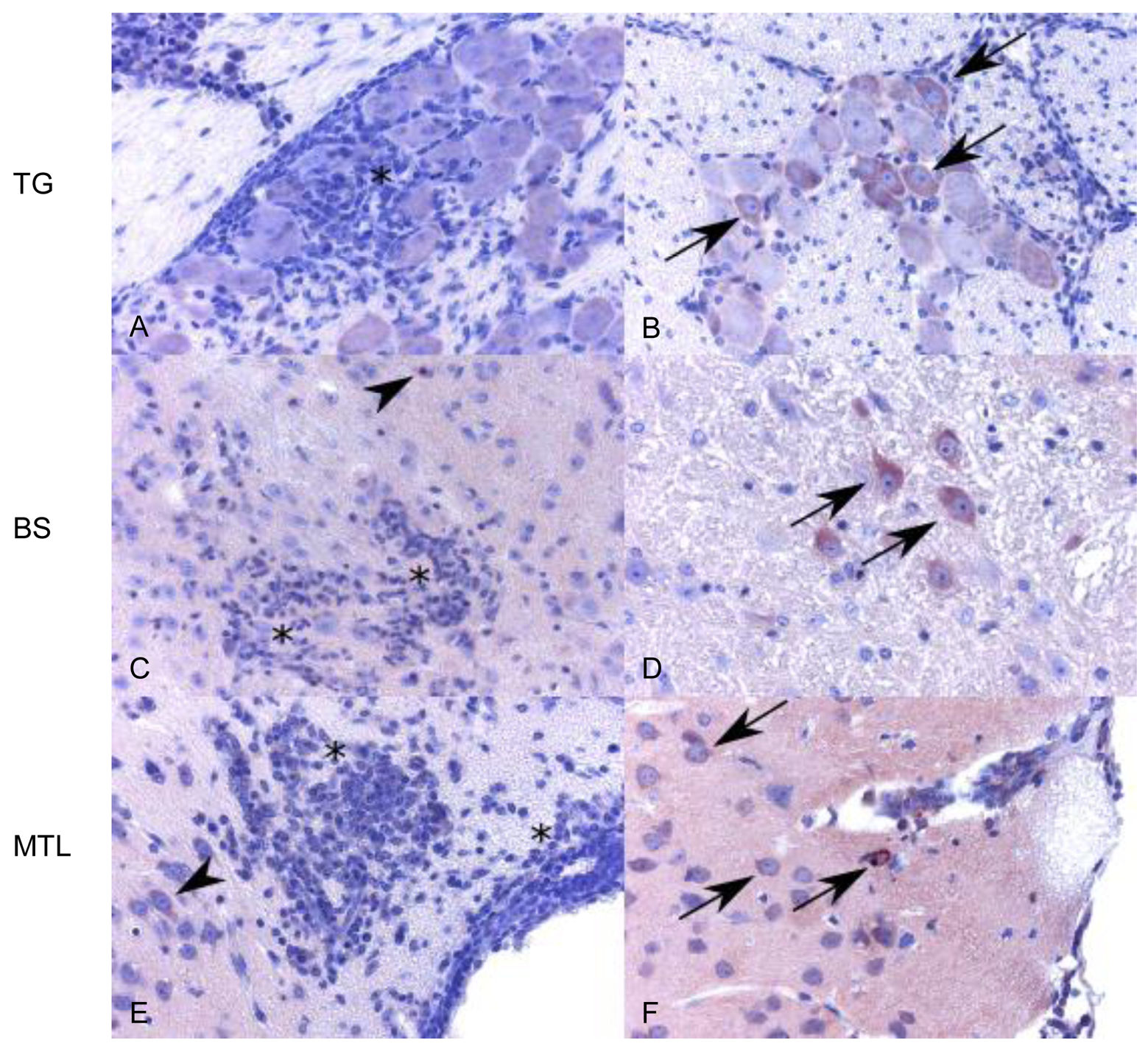

Supplement: S3 Fig — The trigeminal ganglion (TG), brainstem (BS) and mesiotemporal lobe (MTL) were examined for apoptosis. A, C and E) Caspase-3-positive cells only appeared occasionally in regions adjacent to inflammatory spots (asterisk) and included both neuronal and glial cells (arrowhead). B, D and F) Apoptosis of neurons or glial cells was mainly detectable in non-inflamed areas which are indicated by arrows. (TIF) [file ppat.1008445.s005.tif]
